# Changes in oxidation-antioxidation function on the thymus of chickens infected with reticuloendotheliosis virus

**DOI:** 10.1186/s12917-020-02708-6

**Published:** 2020-12-11

**Authors:** Dahan Yang, Chenhui Zhao, Meixi Zhang, Shujun Zhang, Jie Zhai, XueLi Gao, Chaonan Liu, Xiaoping Lv, Shimin Zheng

**Affiliations:** 1 College of Veterinary Medicine, Northeast Agricultural University, 150030 Harbin, People’s Republic of China; 2 Heilongjiang Key Laboratory of Laboratory Animals and Comparative Medicine Harbin, 150030 Harbin, People’s Republic of China; 3 WuXi AppTec (Suzhou)Co., Ltd, 215000 Suzhou, People’s Republic of China

**Keywords:** Reticuloendotheliosis virus, Thymus, Oxidation antioxidant imbalance, Histopathological and ultrastructural changes, Immunosuppression

## Abstract

**Background:**

Reticuloendotheliosis virus (REV) is a retrovirus that causes severe immunosuppression in poultry. Animals grow slowly under conditions of oxidative stress. In addition, long-term oxidative stress can impair immune function, as well as accelerate aging and death. This study aimed to elucidate the pathogenesis of REV from the perspective of changes in oxidative-antioxidative function following REV infection.

**Methods:**

A total of 80 one-day-old specific pathogen free (SPF) chickens were randomly divided into a control group (Group C) and an REV-infected group (Group I). The chickens in Group I received intraperitoneal injections of REV with 10^4.62^/0.1 mL TCID_50_. Thymus was collected on day 1, 3, 7, 14, 21, 28, 35, and 49 for histopathology and assessed the status of oxidative stress.

**Results:**

In chickens infected with REV, the levels of H_2_O_2_ and MDA in the thymus increased, the levels of TAC, SOD, CAT, and GPx1 decreased, and there was a reduction in CAT and Gpx1 mRNA expression compared with the control group. The thymus index was also significantly reduced. Morphological analysis showed that REV infection caused an increase in the thymic reticular endothelial cells, inflammatory cell infiltration, mitochondrial swelling, and nuclear damage.

**Conclusions:**

These results indicate that an increase in oxidative stress enhanced lipid peroxidation, markedly decreased antioxidant function, caused thymus atrophy, and immunosuppression in REV-infected chickens.

## Background

Reticuloendotheliosis (RE) constitutes a group of pathological syndromes caused by reticuloendotheliosis hyperplasia virus, including runting syndrome, chronic tumors of lymphoid and other tissues, acute reticulocytoma, and severe immunosuppression. Reticuloendotheliosis virus (REV) is a C-type avian retrovirus [1, 2]. The REV group includes defective REV-T, non-defective REV-A, chick syncytial virus, duck infectious anemia virus, and spleen necrosis virus (SNV) [3]. In addition, REV infects a wide-range of hosts, including chickens, turkey, duck, goose, Japanese quail, and wild birds, among which turkey is the most susceptible. Thus, turkeys and chickens are commonly used as experimental animals [4–7]. REV can be mixed with Marek’s disease virus (MDV), avian leukosis virus of subgroup J (ALV-J), and chicken anemia virus (CAV), resulting in a reduction or loss of immune function in REV-infected chickens. Thus, RE represents a serious threat to the development of the poultry industry [2, 8].

Under normal physiological conditions, the body’s oxidation-antioxidant system is in a dynamic equilibrium, and oxidative stress occurs when the antioxidant system is overwhelmed by the oxidation system [9, 10]. Oxidative stress is an initial reaction of the body to external stimuli and may subsequently induce various signaling pathways and inflammation in the body [11]. Under oxidative stress, animals grow slowly, the feed conversion rate decreases, and there is a decrease in the production performance [12]. In addition, long-term oxidative stress can deplete antioxidant vitamins and trace elements, impair immune function, as well as accelerate the aging and death of the animal, resulting in substantial economic losses to the poultry industry [13].

The oxidation system primarily includes reactive oxygen species (ROS) and reactive nitrogen species (RNS). ROS and RNS are by-products of the body’s metabolism that contain an unpaired electron. Moreover, ROS and RNS represent an important component of the pathogen resistance mechanism and may act as an intracellular and intercellular signaling molecules; however, excessively high levels may also lead to oxidative stress [14]. ROS primarily includes superoxide anions, hydroxyl radicals, other oxygen radicals, and H_2_O_2_. RNS includes NO, NO_2_, and peroxynitrite [15].

Antioxidant systems include enzymes and non-enzymatic antioxidants [4]. Antioxidant enzymes include SOD, peroxidase, CAT, GPx1, and glutathione reductase, whereas non-enzymatic antioxidants include fat-soluble vitamin E, carotenoids, ubiquinone, and water-soluble vitamin C, glutathione, uric acid, and tryptophan metabolites. It is worthy to mention that imbalance between oxidation-antioxidant system induced excessive ROS and RNS that accumulate and destroy various biomacromolecules (e.g., lipids, proteins, and nucleic acids), which leads to cellular damage and ultimately cell death. Several studies have shown that oxidative stress plays an important role in inflammatory reactions, tumors, and many other diseases [16–18]. It has previously been shown that anti-oxidant treatment in animals can significantly increase the antibody-secretion ability of animals during acute viral infections [19]. Some plant polysaccharides and non-enzymatic antioxidants have a good therapeutic effect on the oxidative stress caused by viral infection [20–22].

Currently, there are few existing studies that have investigated the changes in the oxidation-antioxidant system of poultry following an REV infection. This study examined the dynamic changes in the level of TAC, SOD activity, H_2_O_2_, MDA, CAT, and GPx1, as well as CAT and GPx1 mRNA expression in the thymus of one-day-old SPF chickens after an REV infection. Pathological sections and ultrastructural changes of the thymus were observed to further clarify the pathogenesis of REV infection and morphological changes resulting from oxidative stress and pathological processes, to facilitate the prevention and treatment of RE.

## Results

### Clinical signs and gross lesions in the thymus

**Fig. 1:**
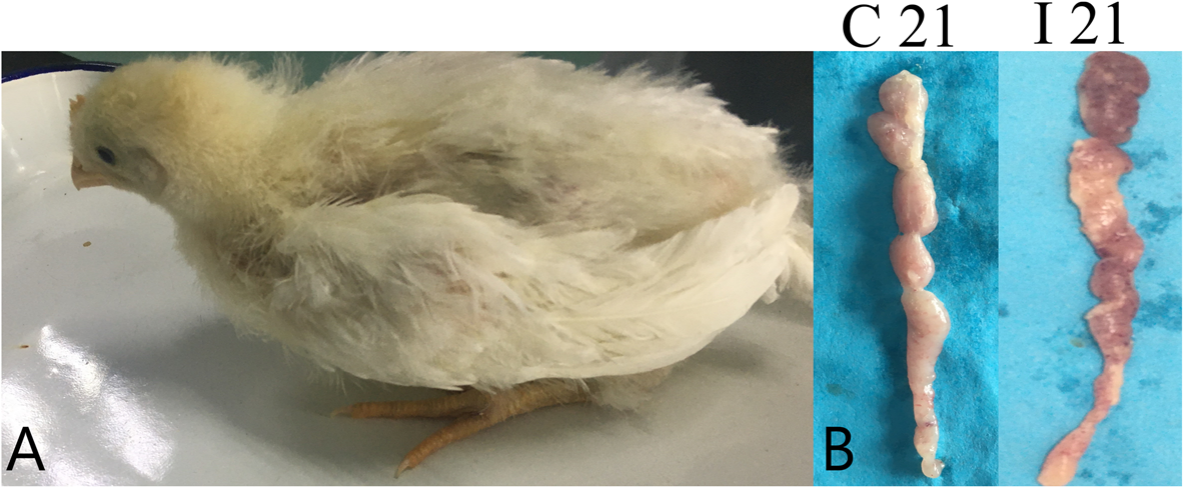
The infected chicken exhibited typical depression. The thymus of Group I and Group C chickens at 21 days post-infection

The chickens in Group I showed obvious reduction in growth, The body weight growth rate was lower than that of the control chickens, and showed typical clinical symptoms (e.g., depression, loss of appetite, and messy feathers). In addition, lesions in the thymus exhibited volumetric reduction and petechial hemorrhages (Fig. 1).

### Determination of oxidative stress biomarkers

**Fig. 2:**
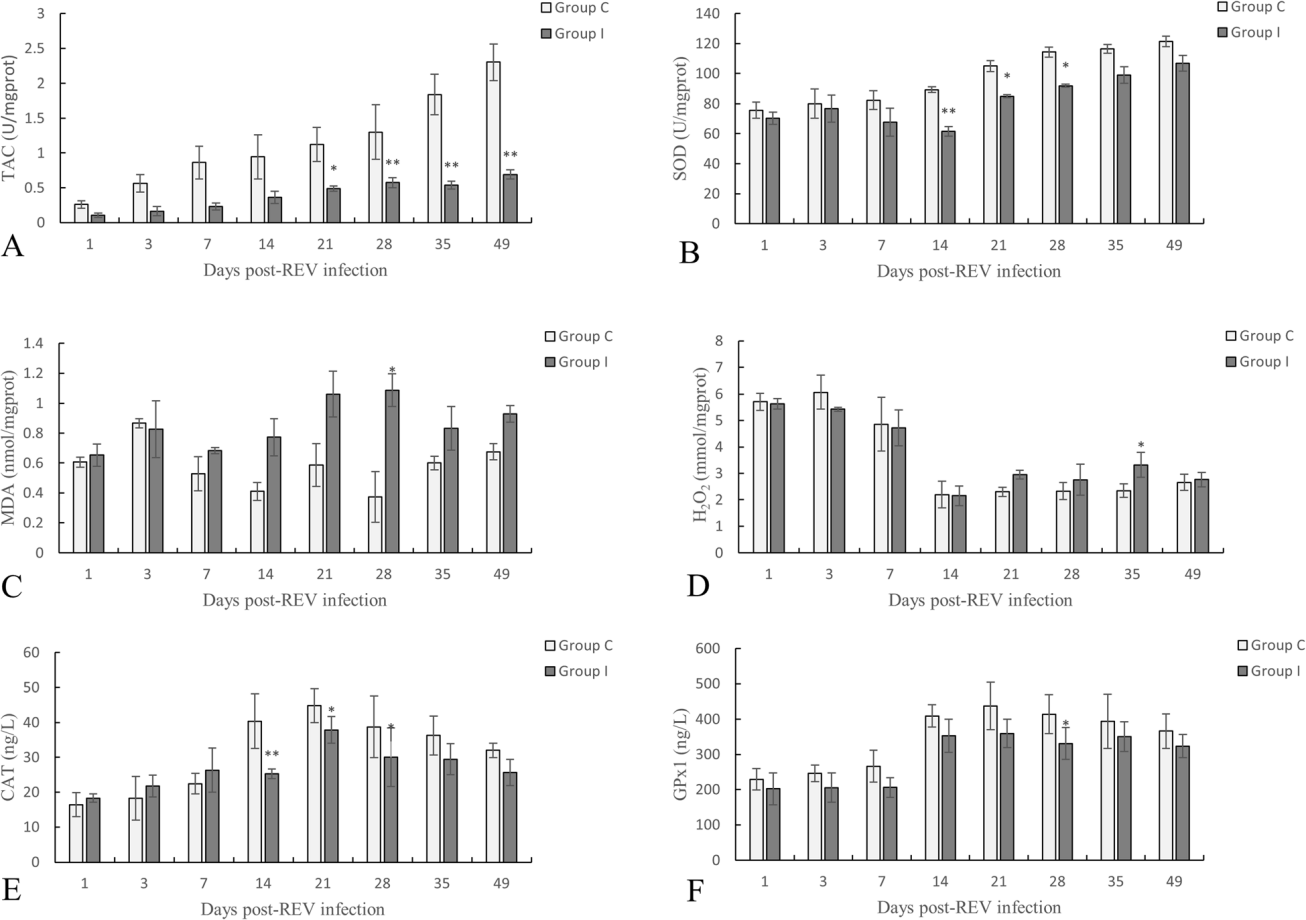
Changes in the oxidative stress biomarkers in chickens infected with REV. **a** The total antioxidant capacity (TAC) of the thymus. **b** The superoxide dismutase (SOD) activity in the thymus. **c** The level of malondialdehyde (MDA) in the thymus. **d** The level of H_2_O_2_ in the thymus. **e** The level of catalase (CAT) in the thymus. **f** The level of glutathione peroxidase 1 (GPx1) in the thymus. Data are presented as the means ± SD (*n* = 5).*(*P* < 0.05) and **(*P* < 0.01) indicates a significant difference when compared to the control

We first sought to study the oxidative-antioxidant capacity of the thymus in chickens infected with REV. We used commercial kits to detect the level of TAC, SOD, H_2_O_2_ activity, and MDA of the chickens in both the infected and control groups. After the chickens were infected with REV, the thymus TAC (Fig. 2a) of the chickens was lower than that of the control chickens. Significant differences were found at 21 (*P < 0.05*), 28 (*P < 0.01*), 35 (*P < 0.01*) and 49 (*P < 0.01*) days post-REV infection. Compared with the control group, the SOD activity (Fig. 2b) of the thymus of the chickens decreased to varying degrees after REV infection, and exhibited significant differences on day 14 (*P < 0.01*), 21 (*P < 0.05*), and 28 (*P < 0.05*) post REV-infection. Moreover, the level of MDA (Fig. 2c) in the thymus of REV-infected chickens increased from day 7 to 49 compared to the control group, and exhibited significant changes on day 28 (*P < 0.05*). The H_2_O_2_ content (Fig. 2d) in the thymus of the chickens increased between 14 and 49 days after REV infection compared to the control group, and showed significant changes on day 35 (*P < 0.05*). Compared with the control group, the level of CAT (Fig. 2e) in the thymus showed a downward trend from day 14 to 49 in REV-infected SPF chickens, and showed significant changes on day 14 (*P < 0.01*), 21 (*P < 0.01*), and 28 (*P < 0.05*). The level of GPx1(Fig. 2f) in the thymus was lower than that of the control SPF chickens after REV infection, and showed significant changes on day 28 (*P < 0.05*) post-infection.

### CAT and GPx1 mRNA expression in the thymus

**Fig. 3:**
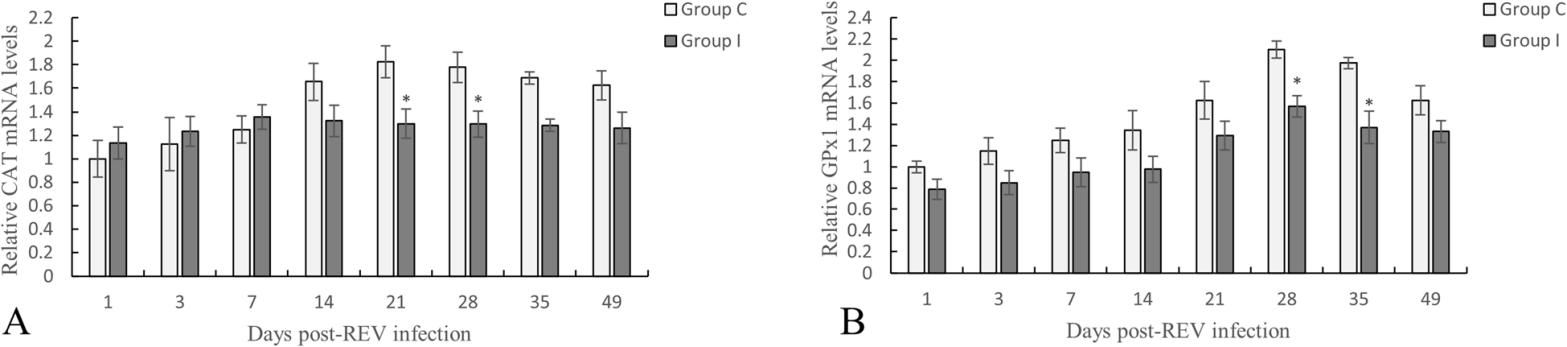
Changes in CAT and GPx1 mRNA expression. **a** CAT mRNA expression in the thymus. **b** Level of GPx1 mRNA expression in the thymus. Data are expressed as the means ± SD (*n* = 5).*(*P* < 0.05) and **(*P* < 0.01) indicates a significant difference compared to the control group

In REV-infected SPF chickens, the level of CAT (Fig. 3a) expression in the thymus decreased from day 14 to 49 compared to the control group, and was significantly decreased on day 21 (*P < 0.05*) and 28 (*P < 0.05*). From day 1 to 49 post-REV infection, the level of GPx1 expression (Fig. 3b) in the thymus of SPF chickens in the infected group was lower than that in the control chickens. Whereas, significant changes were observed on day 28 (*P < 0.05*) and 35 (*P < 0.05*).

### Determination of the Thymus/BW index

**Fig. 4:**
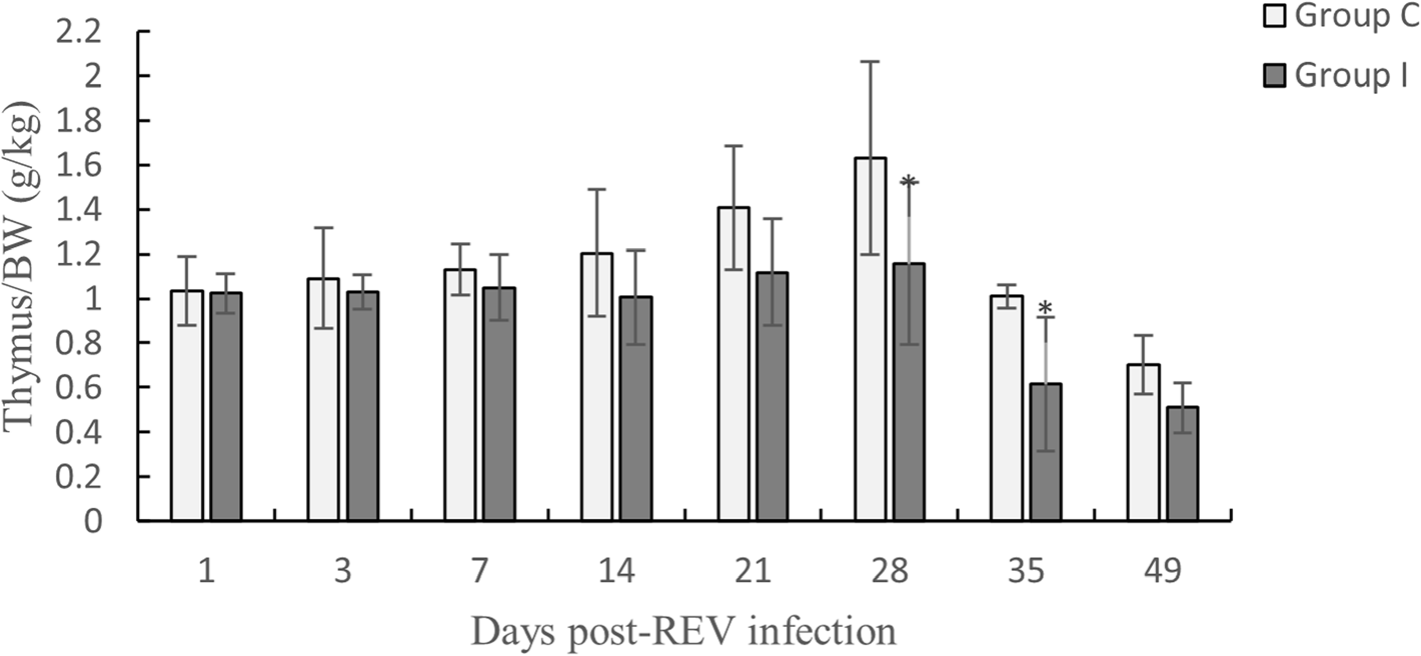
Changes in the Thymus/BW index. Data are expressed as the means ± SD (*n* = 5).*(*P* < 0.05) and **(*P* < 0.01) indicate a significant difference compared to the control group

In SPF chickens infected with REV, the Thymus/BW index (Fig. 4) was lower than that of the control chickens, and was significantly lower than the control chickens on day 28 (*P < 0.05*) and 35 (*P < 0.05*).

### Histopathological changes in the thymus of REV-infected SPF chickens

**Fig. 5:**
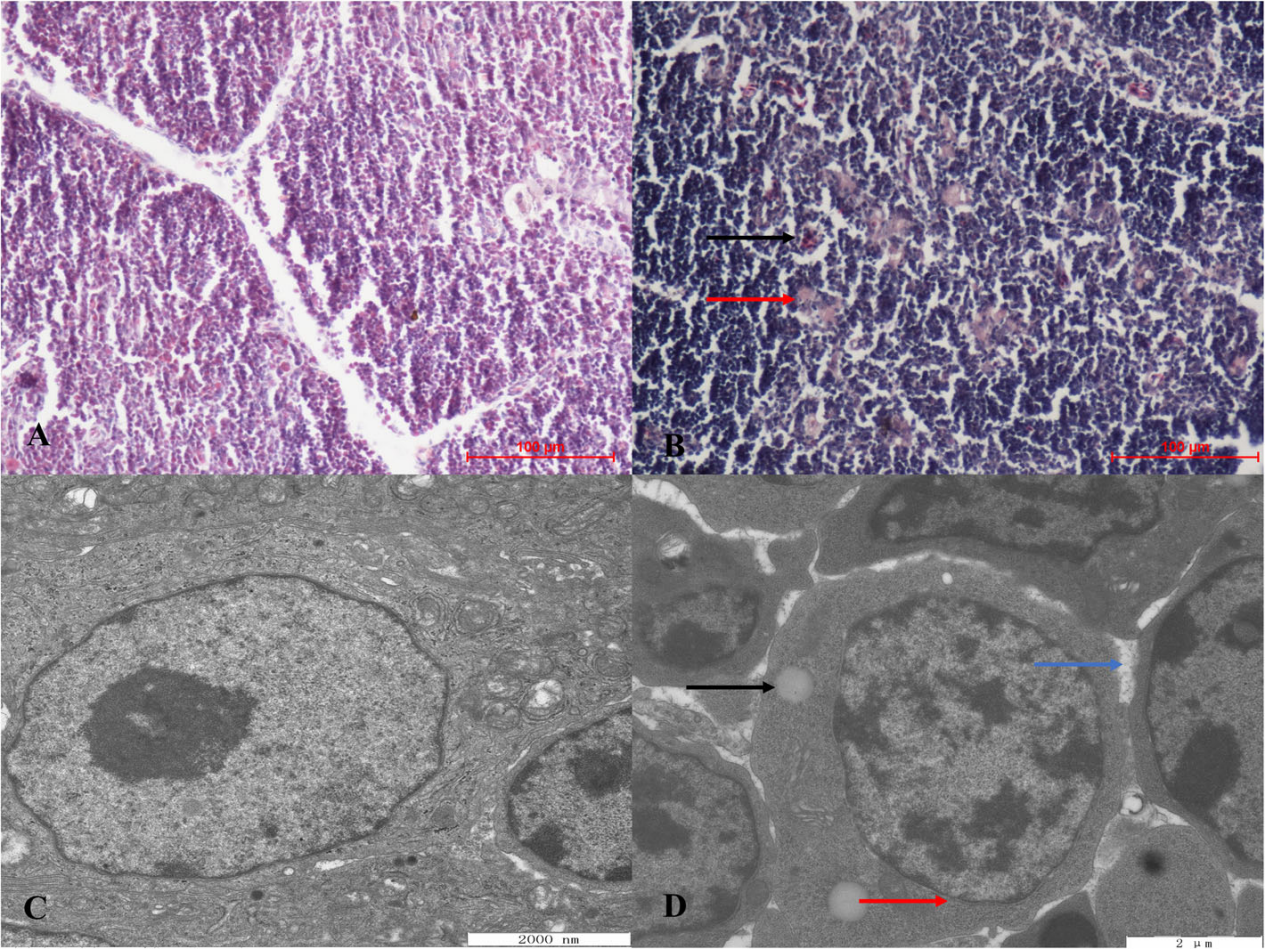
Histological and ultrastructural changes in the chicken thymus after REV infection. Histological examination of the thymus in the control group (**a**) and REV infection group (**b**) of chickens by HE staining (200x). Histological results showed significant necrosis (↑) and haemorrhage (↑) in the REV infection group. Transmission electron microscopy examination of the thymus in the control group (**c**) and REV infection group (**d**) of chickens (15 000x).Ultrastructural results showed that the REV infection group showed nuclear membrane rupture (↑), mitochondrial vacuolization (↑) and enlarged intercellular space (↑)

The histomorphology and ultrastructural changes of the thymus are presented in (Fig. 5). Compared with the control group, the thymic lobule was atrophied, the cortex and medulla exhibited hemorrhaging, and there was narrowing of the cortical area. In addition, there was a reduced number of lymphocytes in the medulla, an increased number of reticuloendothelial cells, and enhanced inflammatory cell infiltration. The ultrastructure of the control group appeared normal under electron microscope. However, there was obvious cell damage, which primarily manifested as mitochondrial swelling, sputum rupture, Golgi vesicle expansion, and decreased cytoplasm in the REV-infection group. In addition, the nuclear membrane was ruptured and dissolved, and the nucleolus was enlarged.

## Discussion

Several studies demonstrated that ROS activated the immune system to eliminate the infection and played a positive regulatory role in viral infections [23, 24]. Simultaneously, the virus breaks the oxidation-antioxidant balance of the host cells, causing the accumulation of excessive amount of ROS, which results in cellular damage and apoptosis [25]. RE is an immunosuppressive tumor disease caused by REV. In the present study, we examined changes in the oxidative-antioxidative function of the thymus in chickens after REV infection. It was found that REV can cause oxidative stress in the thymus and reduce antioxidant function.

Under conditions of oxidative stress, ROS and RNS promote the accumulation of lipid peroxidation in polyunsaturated fatty acids in oxidized biofilms, and produce lipid peroxides (e.g., MDA, ketone, hydroxyl, and carboxyl) [26]. MDA can reflect the degree of lipid peroxidation in the body and can be used as an indicator to determine whether a cell is oxidatively damaged [27, 28]. In this study, the MDA content in the thymus was significantly higher on day 28 in REV-infected chickens, compared to that of the control chickens. H_2_O_2_ is an important ROS that can be scavenged by catalase or peroxidase, and can also react with free Fe^2+^ to produce ·OH to initiate lipid peroxidation, destroy the cell membrane structure, accelerate protein decomposition and results in nucleic acid damage [29, 30]. In this study, the level of H_2_O_2_ in the thymus on day 35 in REV-infected chickens was significantly higher than that of the control chickens. Our research shows that the level of biomarkers of oxidative stress increase in REV-infected chickens. In our previous study, REV infection caused thymic lymphocyte apoptosis, inhibited T cell proliferation, and enhanced the immunosuppressive effect [31]. Moreover, Qi et al. showed that avian influenza virus H9N2 can induce chicken oviduct epithelial cell apoptosis by ROS accumulation and mitochondria-mediated apoptosis signaling pathway [32]. Accordingly, we speculate that REV infection induces oxidative stress in the chicken thymus to produce a large amount of ROS and RNS, which may be the cause of thymic lymphocyte apoptosis.

Under normal conditions, SOD can catalyze the disproportionation of O_2_^•−^ in the body to produce both H_2_O and H_2_O_2_ [14]. H_2_O_2_ can break the structure and function of the cell membrane, react with purines and pyrimidines, and result in nucleic acid damage [30]. Viral infection typically stimulates the production of ROS and inhibits antioxidant enzyme activity [33]. CAT can remove excess H_2_O_2_ in the body, and excess -OOH can be reduced to -OH under the action of GPx1. The main function of GPx1 is to remove lipid peroxides; however, in organs or tissues with low H_2_O_2_ production or CAT content, it can replace CAT to remove H_2_O_2_. GPx1 can scavenge lipid peroxides to reduce damage to the body and play a positive role in oxidative defense reactions [34]. CAT and GPx1 work together to clear H_2_O_2_ from the body [11]. Therefore, we evaluated the activity of these enzymes in the thymus of chickens infected with REV. This study found that REV-infected SPF chickens exhibited lower levels of SOD activity, CAT, GPx1 content, as well as CAT and GPx1 mRNA expression, and increased biomarkers of oxidative stress (e.g., MDA and H_2_O_2_) compared to the control group. In addition, we also evaluated TAC and found that the level of TAC in the thymus was significantly decreased in the infected group. This finding indicated that the antioxidant capacity of the thymus decreases during the REV infection, and excess ROS cannot be effectively neutralized by the antioxidant enzymes, which leads to oxidative stress.

Oxidative stress is related to viral pathogenesis. ROS production is caused by inflammatory cell activation (e.g., neutrophils) during the course of disease progression and is considered to be an important defense against the host virus. However, excessive ROS can have an adverse effect on the body [35]. In avian viral infectious disease research, some scholars have found that iNOS plays an important role in the pathogenic mechanism of highly pathogenic avian influenza infection in mice, and antioxidant therapy can effectively regulate the immune response of the host [36, 37]. In addition, Newcastle Disease Virus (NDV) can significantly increase the level of NO and MDA in the intestines of chickens, and significantly reduce the activity of GST, CAT, SOD, and TAC. However, the addition of vitamin E to the diet can significantly reduce the level of intestinal oxidative stress in chicks and decrease the impact of viral infections on chickens [38, 39]. Some previous studies reported that Sargassum polysaccharides can resist IBDV infection by improving the antioxidant capacity and cytokine levels of chicken bursal lymphocytes [22]. This indicates that many poultry virus infections can induce oxidative stress in the body, and some Chinese medicinal extracts have significant therapeutic effects in the antioxidant treatment of poultry diseases. This study found that REV-infected chickens have lower levels of CAT, SOD, and GPx1 content, and increased MDA content compared to the control group. These findings indicate that REV infection can lead to a decrease in the level of blood antioxidants and an increase in oxidation and cause of oxidative stress in chickens. This is consistent with the results of this study on the changes in the oxidative and antioxidant capacity of the thymus of REV-infected chickens [40]. Combined with the results of this study, the growth inhibition and pathological damage in the thymus caused by REV may be related to an accumulation of oxidizing substances and a decline in antioxidant system functionality.

## Conclusions

Following REV infection in one-day-old SPF chickens, the content of MDA and H_2_O_2_ and superoxide in the thymus was increased, the TAC and SOD activity decreased, and the Thymus/BW index was reduced. These changes exceeded the body’s ability to scavenge antioxidant substances, resulting in oxidative stress in the body. In the future, we aim to study the effects of REV on other physiological mechanisms in the thymus from the perspective of endoplasmic reticulum stress and apoptosis based on the results of oxidation and antioxidant research.

## Methods

### Experimental birds and REV strain

All of the chickens used for experiments were one-day-old SPF chickens obtained from Harbin Veterinary Research Institute (HVRI), Chinese Academy of Agricultural Sciences (CAAS), China. The REV-T strain (CVCC No. CACCAV107) was purchased from China Veterinary Culture Collection Center (CVCC). The virus TCID_50_ was 10^4.62^/0.1 mL as determined by cell breeding.

### Experimental design and sample collection

A total of 80 one-day-old SPF chickens were randomly divided into a control group (Group C) and an REV-infected group (Group I). Group I were intraperitoneally administered with 100 µL of viral suspension at day 1. Group C were intraperitoneally administered 100 µL sterile physiological saline. The two groups of chickens were housed in isolators with similar environments, and the chickens were given free access to feed and water. Each group further consist of 8 cages (5 chickens/cage). In order to reduce the artificial error and ensure the repeatability of the experiments, we decided to set the sample size for each test index to 5 chickens, as described previously [41, 42]. During housing, animals were monitored twice daily for the health status and no adverse events were observed. On day 1, 3, 7, 14, 21, 28, 35, and 49 post-REV infection, a cage of chickens were randomly selected from each group and euthanasia was performed by sedation using a Rompun/Ketamine(1 mg/kg) mixture as an intramuscular thigh injection followed by an intravenous wings injection of Pentobarbitone (150 mg/kg). The thymus was dissected and homogenized in chilled sterile physiological saline using a glass homogenizer with a Teflon pestle under cold conditions. The homogenate was centrifuged at 4000 × g for 10 min at 4 °C. The supernatant was used for the assessment of oxidative stress biomarkers.

### Determination of thymic oxidative stress biomarkers

The prepared 10% tissue homogenate was removed from − 80 °C. The level of TAC, SOD, H_2_O_2_ activity, and MDA in the thymus was determined using commercially available detection kits (Nanjing Jiancheng Bioengineering Research Institute, China). The level of CAT and GPx1 was measured by an ELISA, according to the manufacturer’s instructions (Qiyi Biological Technology Co., Ltd. China).

### Total RNA extraction and detection

The total RNA in the thymus was extracted by using Trizol reagent (Invitrogen, Shanghai, China). The quality of RNA was assessed using a NanoDrop2 000 spectrophotometer (Thermo Fisher Scientific) through the ratio of absorbance at 260 nm and 280 nm. cDNA was obtained by reverse transcription of the RNA using the M-MLV Reverse Transcriptase kit (Invitrogen) according to the manufacturer’s instructions.

### Real-time PCR

Determination of CAT and GPx1 mRNA in the thymus by real time PCR. The CAT, GPx1, and β-actin primers were designed using Primer5.0 software. CAT: forward primer 5’- ATGTCCGTTTCAGGAGATGTGCAGC-3’, reverse primer 5’- CCAGCAGTGCCTGAATACG − 3’; GPx1: forward primer 5’- ATGACCAACCCGCAGTACATCATCT-3’, reverse primer 5’- GCAGTTTGATGGTCTCGAAGTGGC-3’; β-actin forward primer 5’- CGGGACGGATGAGAAGAA − 3’, reverse primer 5’- TCGGCGCTCCAGATGTAC − 3’. Amplification of the target gene using the LightCycler2.0 real-time PCR System (Roche480, USA). The real-time PCR reaction procedure was 95 °C for 2 min, 45 cycles of 95℃ for 20 s, 59℃ for 20 s, and 72℃ for 10 s. To ensure the repeatability of the amplified samples, all samples were analyzed in triplicate. β-actin was used as a reliable normalization gene, and the expression of the samples were evaluated in relation to this housekeeping gene. The level of CAT and GPx1 mRNA expression were analyzed in accordance with the 2^−ΔΔCt^ method.

### Determination of the thymus index

Weight of the chickens and thymus at the time of sampling for calculating the thymus index. The thymus index was calculated based on the weight of the thymus (g)/chicken weight(kg).

### Pathological changes of the thymus following REV infection

Thymus tissues were fixed in 4% formalin. The samples were dehydrated with ethanol and embedded in paraffin. Each sample was cut into 5-µm sections with a microtome and placed on a glass slide, and stained with hematoxylin and eosin (H&E). The pathological changes of the thymus were observed by three pathologists unaware of the experimental conditions under both light microscope (Nikon, H600, Japan) and electron microscope (Hitachi 7650, Tokyo, Japan).

### Statistical analyses

The data were analyzed with SPSS 17.0 software, and the differences were compared between the groups were analyzed using a one-way ANOVA followed by a Tukey’s post-hoc test. All data were expressed as the mean ± SD. *P < 0.05* was considered as statistically significant.

## Data Availability

The datasets used and analysed during the current study are available from the corresponding author on reasonable request.

## Abbreviations

REV: Reticuloendotheliosis virus
SPF: Specific pathogen free
MDA: Malondialdehyde
TAC: Total-antioxidant capacity
SOD: Superoxide dismutase
ELISA: Enzyme linked immunosorbent assay
CAT: Catalase
GPx1: Glutathione peroxidase 1
ROS: Reactive oxygen species
RNS: Reactive nitrogen species
